# Small Vessel Disease

**DOI:** 10.3389/fneur.2019.01020

**Published:** 2019-09-24

**Authors:** Antoine M. Hakim

**Affiliations:** ^1^Ottawa Hospital Research Institute, Ottawa, ON, Canada; ^2^Faculty of Medicine, Brain and Mind Research Institute, University of Ottawa, Ottawa, ON, Canada; ^3^Division of Neurology, University of Ottawa, Ottawa, ON, Canada

**Keywords:** SVD, vascular risk factors, inflammation, dementia, lifestyle

## Abstract

Small vessel disease (SVD) refers to conditions where damage to arterioles and capillaries is predominant, leading to reduced, or interrupted perfusion of the affected organ. Data suggest that when this condition is evident in any organ, it is already systemic in its occurrence and consequences. SVD affects primarily organs that receive significant portions of cardiac output such as the brain, the kidney, and the retina. Thus, SVD is a major etiologic cause in debilitating conditions such as renal failure, blindness, lacunar infarcts, and dementia. The factors that lead to this devastating condition include all the known vascular risk factors when they are not strictly controlled, but lifestyles that include sedentary existence, obesity, and poor sleep patterns are also recognized drivers of SVD. In addition, depression is now recognized as a vascular risk factor. Inflammation is a mediator of SVD, but it is not known which factor(s) predominate in its etiology. This article emphasizes the need for more investigations to define this link further and suggests clinical and societal responses that might reduce the major impacts of this condition on populations.

## Introduction

Small vessel disease (SVD) refers to any pathologic process that damages small end arteries, arterioles, venules, and brain capillaries (1). Although this condition was described pathologically more than a century ago (2), its pervasive nature and its association with clinically relevant conditions have been increasingly appreciated as imaging technologies advanced and became more accessible (3).

## Pathology and Pathophysiology of SVD

The main target of SVD is the endothelium, the organ that is a barrier between circulating blood and the vessel wall. Healthy endothelial cells control vascular permeability to components in plasma, limit aggregation of platelets and leucocytes, and regulate vascular tone, all of which is essential if blood flow will match metabolic demand (4). The main task of the endothelial cells is therefore to integrate information scanned constantly from the vessel wall, the blood flowing through the arterioles, and from the physiological parameters defining vascular health, and respond by varying vasomotor tone in the arterioles and maintaining an adequate antithrombotic surface.

While the endothelial cells are the main contributors to the properties of the blood brain barrier, their capabilities are induced and maintained by their interactions with mural cells (pericytes and vascular smooth muscle cells), immune cells, and glial cells—predominantly astrocytes (5). As a result, SVD results in lipohyalinosis and arteriolosclerosis.

Several animal models have been studied to shed light on the physiological processes leading to and accompanying SVD, and most involve sustained hypoperfusion of the brain as a result of bilateral stenosis of the common carotid arteries (6). This results in multiple small infarcts and hemosiderin-like regions in subcortical nuclei, with the eventual appearance of cerebral atrophy.

## Inflammation and SVD

There is a growing appreciation that endothelial cells may become damaged when the environment is inflammatory (7). Inflammatory markers have been associated with periventricular white matter hyperintensities (WMH), the main MRI signature of SVD in the brain (8). Reports have linked a number of abnormalities in the interleukin (IL) pathways to SVD including IL-1beta, tumor necrosis factor, IL-10, IL-21, and IL-23 (9), confirming earlier findings by Flex et al. linking proinflammatory gene polymorphisms to one of the major consequences of SVD, dementia (10). C-reactive protein (CRP), a non-specific marker of inflammation, is considered an endothelial toxin, and CRP is both predictive of SVD and is elevated when the condition is evident (11). A relationship between midlife systemic inflammation and late-life SVD has also been reported (12).

## Neuroimaging of SVD

The damage caused by SVD impacts both deep gray and white matter structures in the brain (13). While CT scans can reveal the presence of SVD, the ideal method for visualizing the full spectrum of SVD is structural MRI. This includes FLAIR, T1 and T2-weighted as well as gradient echo MRI sequences (14). More recently, whole-brain magnetic resonance spectroscopy has also been used to study markers of brain inflammation (15). These brain imaging modalities reveal in SVD recent small subcortical infarcts, hyperintensities in the periventricular and deep white matter, lacunes, microbleeds, and enlarged perivascular spaces, located predominantly in the basal ganglia and the subcortical white matter (16). As well, the blood-brain barrier is impaired in small vessel disease (17). Eventually, brain atrophy becomes evident (18). Combining MRI studies with measurements of cerebral blood flow and metabolism using positron emission tomography is allowing new insights linking the progression of physiological and imaging abnormalities to the development of cognitive deficits (19, 20).

## Health Consequences of Small Vessel Disease

In 2016 a report from experts in the field sponsored by the National Institutes of Neurological Diseases and Stroke titled: “Small Blood Vessels: Big Health Problems,” highlighted the systemic nature of the disease and its neurological consequences (21). The current review unequivocally confirms the conclusion that the presence of SVD in any organ implies that the patient is suffering from a “systemic” condition.

In the brain, SVD is strongly associated with stroke (22), and the presence of SVD in the brain hampers recovery in patients who have suffered a stroke (23). SVD in the brain is also associated with declines in psychiatric (24), and gait functions (25). As might be expected from these statements, and as already noted, SVD is a major etiologic factor in dementia (26).

If brain imaging modalities reveal SVD, there is a high probability that the condition is also affecting in variable combinations the retina (27), the kidneys (28–31), the heart (32), the lungs (33), and the musculoskeletal system (34)—in fact, every organ that is perfused at a high rate of flow. Although it is possible that all these organs are impacted by the same vascular risk factors, there is nonetheless multi-organ involvement with SVD, and the link appears multidirectional. Thus, it was reported recently that retinal neurodegeneration can predict cognitive impairment (35, 36), and small vessel disease as well as retinal microvascular abnormalities are now confirmed to be associated with an increased risk of renal failure (30, 31). Consequently, in addition to damaging the brain, SVD is perhaps the main etiology for blindness, renal dysfunction, and cardiac insufficiency, making it the etiology for major medical, and neurological conditions.

## Who is a Candidate For SVD?

A paper published in 2007 by Vermeer et al. outlined the conditions associated with MRI-defined silent brain infarcts (37). The highest associations were noted with prior stroke, cardiovascular risk factors, and the presence of renal failure (37). This article also identified depression as having a strong association with silent brain infarcts, a link that has been confirmed subsequently (38). A 2014 study that specifically tested the association of total SVD score with vascular risk factors confirmed that smoking, hypertension, male sex, and advancing age were conditions that promoted brain SVD (39). In a separate study, SVD was observed in 3% of 40–49-year-olds but 18.9% of 70-year-olds, highlighting the association of SVD with aging (40).

In terms of the cardiovascular risk factors, hypertension is a major driver of SVD (41), and ambulatory measurements of blood pressure are better predictors of WMH than self-reported measurements (42). In the setting of hypertension, the risk of accumulating WMH rises rapidly above a resting systolic blood pressure of approximately 120 mmHg (43). Systolic blood pressure sustained above this threshold also predicts a growing risk for dementia and strokes (42, 44). Despite this overwhelming evidence in favor of maintaining at-rest systolic blood pressure at 120 mmHg, concern is often expressed that this may lead to increased mortality in the elderly, perhaps contributed to by falls and hip fractures due to orthostatic hypotension and dizziness (45). The implications of these new guidelines and the challenges they represent merited an entire issue of Circulation Research (46). It is important to note that in the SPRINT trial, among patients aged 75 or older, primary outcomes of myocardial infarction, acute coronary syndrome, stroke, congestive heart failure and cardiovascular death were all significantly lower in those whose systolic blood pressures were maintained at or below 120 mmHg (47). Despite this, Canada's hypertension guidelines still define hypertension as systolic blood pressure of more than 135 mm Hg (48).

Obesity's impact on the development of brain small vessel disease is now well-documented (49). A 2017 review concluded that obesity and its comorbidities were associated with impaired cognitive performance, accelerated cognitive decline and dementia in later life (50), confirming the conclusion of a systematic review by Pedditzi et al., published a year earlier (51). In the setting of obesity there is a significant rise in the factors promoting inflammation, leading to the conclusion that accumulated fat, particularly in its abdominal location, is a source for inflammatory cytokines (52). It is perhaps not surprising therefore that obesity has been associated with smaller brain volumes (53).

Another reason for referring to obesity's negative impact on brain function is its high prevalence in our societies. In the United States, in 2010, 35.5% of men and 35.8% of women satisfied the criteria for obesity (Body Mass Index (BMI) of 30 or more) (54), with a clear trend for increasing prevalence of abdominal obesity between 1999 and 2012 in both men and women (55). In 2009, 24% of Canadian men and women satisfied the criteria for obesity (56).

Several other vascular risk factors have been associated with greater occurrence of SVD. These include prediabetes (57), untreated diabetes (58, 59), and hyperlipidemia (60, 61). In diabetes, microvascular complications are associated with proinflammatory cytokines and with both vascular and intracellular cell adhesion molecules (62). It is now also clear that gut microbiota can impact brain health through immune influences that can lead to cerebral endothelial dysfunction. In this context, new evidence suggests that dietary salt may promote neurovascular and cognitive dysfunction through a gut-initiated TH17 response (63).

A newly emphasized risk factor for SVD is a poor sleep pattern (64). Those who chronically cheat themselves out of sufficient sleep suffer from increased systemic inflammation (65) and are at risk for SVD and eventually develop cortical atrophy (66). The most likely reason for this is that metabolic clearance from the brain occurs preferentially during sleep (67). Unfortunately, it is estimated that a large segment of the population lives daily with inadequate sleep (68), particularly in school aged children (69).

As noted above, depression was identified by Vermeer as a factor promoting silent brain infarcts (37), and depression has been confirmed to be associated specifically with SVD (70, 71). The importance of this association arises from the disturbing statistics about the prevalence of loneliness in society, a state of mind that is highly correlated with depression (72). In the United States, it has been estimated that 40% of Americans felt lonely (73), and a survey by Statistics Canada in 2012 reported that 20% of older people declared feeling lonely (74). Thus, several societal factors associated with loneliness and depression may be promoting SVD.

## SVD and the Risk for Dementia

Since the prevalence of SVD increases with age, it is the consensus that the prevalence of dementia will increase over time as life expectancy lengthens (75). SVD is associated with both a reduction in cerebral blood flow (CBF) (76), and impaired CBF autoregulation (77), making it a key player in the etiologic mix that leads to dementia. Alzheimer's disease, where beta-amyloid and tau proteins accumulate in the brain, does lead to dementia, but the genetically inherited Alzheimer's variant becomes apparent early and impacts patients in their 50's and 60's. When individuals live past that age with normal cognitive function, the physiological profile that leads to dementia is very different. As mentioned above, data from the ADNI cohort (Alzheimer's Disease Neuroimaging Initiative) have clearly shown that when individuals are still cognitively intact, the sequence of pathophysiologic events that eventually leads to a decline in cognitive function starts with a reduction in cerebral blood flow (20), an association that was confirmed in a population-based study (78). This results in both reduced energy supply and reduced ability to clear accumulated waste. The important point to remember is that the reduction in CBF occurs before glucose dysregulation, before the abnormal accumulation of A-beta and other molecular biomarkers in the CSF, and before any impairment in cognition is evident. This new appreciation for the role of cerebral blood flow in delivering energy to the brain and removing the accumulated toxic molecules is perhaps one explanation for the failure of drugs designed to improve symptoms of cognitive decline by reducing the cerebral load of beta-amyloid (79, 80). Consequently, protecting CBF from decline and securing the brain's energy supply are crucial to our ability to defend against dementia.

The powerful message in all of this is that we need to explore the premise that individuals and societies may succeed in lowering their risk for dementia by reducing vascular risk factors and changing potentially harmful lifestyles. A review of advances in stroke research accomplished during 2018 confirms the impact of adverse lifestyle exposures on brain health and on cognition (81). Decades ago the lesson was established that a successful cure is dwarfed in its societal impact by a reduction in risk, and books intended for the public are now available with advice on how to achieve the goal of reducing the risk for dementia (82, 83).

## SVD Deserves More Research Attention

Because SVD represents a major and growing threat to vascular health, it deserves more focused research attention. Addressing the specific pathophysiology of SVD is urgently needed. Several endothelial toxins associated with SVD are already known (84), but if we knew which played predominant roles, then it might be possible to test in SVD therapeutic tools to decrease the concentration of the most harmful inflammatory molecules. Another approach may arise from our knowledge that sustained activation of certain pro-angiogenic signaling factors studied in cancer biology can promote the generation of new blood vessels (85). Could one or more of these factors be tested as repair paradigms in the setting of SVD?

## Ideal Response to SVD at the Clinical Level

The patient with SVD keeps many subspecialists occupied. He or she sees the neurologist for failing memory function and for other consequences of lacunar infarcts, the ophthalmologist for failing vision, the nephrologist for failing kidney function, the cardiologist for heart failure, and the family physician and geriatrician for control of vascular risk factors. Each specialist is focused on how to protect his or her subspecialty organ from damage. As a result, there is enormous duplication of effort, when a centralized “Vascular Clinic” that focusses on optimizing the function of the small blood vessels may be effective. In addition to monitoring and treating the vascular risk factors, such a clinic would also help patients with the lifestyle changes they need to bring about to reduce further SVD and provide support to accomplish these goals.

## Ideal Societal Response

SVD puts a very heavy burden on our individual and national health care budgets. Add to that the unpaid labor of caregivers to patients afflicted by the consequences of SVD, and the cost increases further. Society needs to respond with a cohesive and integrated set of actions.

When we became convinced that smoking was the cause of some lung cancers, clinicians, and scientists developed a discourse with our patients and the public emphasizing the serious consequences of smoking. Eventually society took on the industry—and it was not easy. To reduce the incidence of SVD we need to regenerate that energy, educate the public by talking about the damaging consequences of untreated high blood pressure and all the other vascular risk factors we know. Like there was in smoking, there are industries that will oppose us. Focused taxation may be one solution as it is now proven to limit the consumption of items known to contribute to SVD (86).

## Conclusion

We now recognize that small vessel disease is a major health threat, aggravated by the current lifestyle trends in our society. We need to understand the disease better and mobilize energies to improve its prevention and treatment.

## Author Contributions

The author confirms being the sole contributor of this work and has approved it for publication.

### Conflict of Interest

The author declares that the research was conducted in the absence of any commercial or financial relationships that could be construed as a potential conflict of interest.
